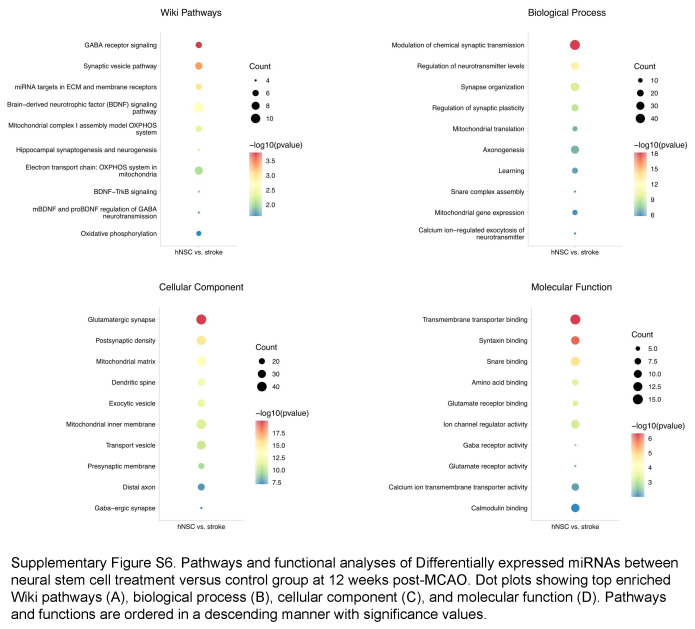# Stereotactically intracerebral transplantation of neural stem cells for ischemic stroke attenuated inflammatory responses and promoted neurogenesis: an experimental study with monkeys

**DOI:** 10.1097/JS9.0000000000001791

**Published:** 2024-06-14

**Authors:** Yi-Fan Liu, Hao-Tian Liu, Chuheng Chang, Cheng-Xian Yang, Xin-Nan Liu, Xia Wang, Wei Ge, Ren-Zhi Wang, Xin-Jie Bao

**Affiliations:** a Department of Neurosurgery, Peking Union Medical College Hospital, Chinese Academy of Medical Sciences and Peking Union Medical College, Beijing; b Department of Neurosurgery, West China Hospital, Sichuan University, Chengdu, Sichuan; c Department of Immunology, Institute of Basic Medical Sciences, Chinese Academy of Medical Sciences, School of Basic Medicine, Peking Union Medical College, Beijing; d Department of Radiation Oncology, National Cancer Center/Cancer Hospital, Chinese Academy of Medical Sciences and Peking Union Medical College, Beijing; e Department of Orthopaedics, Peking University First Hospital, Beijing; f School of Medicine, Life and Health Sciences, The Chinese University of Hong Kong, Shenzhen, Guangdong; g State Key Laboratory of Common Mechanism Research for Major Diseases, Beijing, China

**Keywords:** cynomolgus monkeys, human neural stem cells, ischemic stroke, micro-RNA, proteomes

## Abstract

**Background:**

Ischemic stroke is a common neurovascular disorder with high morbidity and mortality. However, the underlying mechanism of stereotactically intracerebral transplantation of human neural stem cells (hNSCs) is not well elucidated.

**Materials and methods:**

Four days after ischemic stroke induced by Rose Bengal photothrombosis, seven cynomolgus monkeys were transplanted with hNSCs or vehicles stereotactically and followed up for 84 days. Behavioral assessments, magnetic resonance imaging, blood tests, and pathological analysis were performed before and after treatment. The proteome profiles of the left and right precentral gyrus and hippocampus were evaluated. Extracellular vesicle micro-RNA (miRNA) from the peripheral blood was extracted and analyzed.

**Results:**

hNSC transplantation reduced the remaining infarcted lesion volume of cynomolgus monkeys with ischemic stroke without remarkable side effects. Proteomic analyses indicated that hNSC transplantation promoted GABAergic and glutamatergic neurogenesis and restored the mitochondrial electron transport chain function in the ischemic infarcted left precentral gyrus or hippocampus. Immunohistochemical staining and quantitative real-time reverse transcription PCR confirmed the promoting effects on neurogenesis and revealed that hNSCs attenuated post-infarct inflammatory responses by suppressing resident glia activation and mediating peripheral immune cell infiltration. Consistently, miRNA-sequencing revealed the miRNAs that were related to these pathways were downregulated after hNSC transplantation.

**Conclusions:**

This study indicates that hNSCs can be effectively and safely used to treat ischemic stroke by promoting neurogenesis, regulating post-infarct inflammatory responses, and restoring mitochondrial function in both the infarct region and hippocampus.

## Graphical abstract

**Figure:**
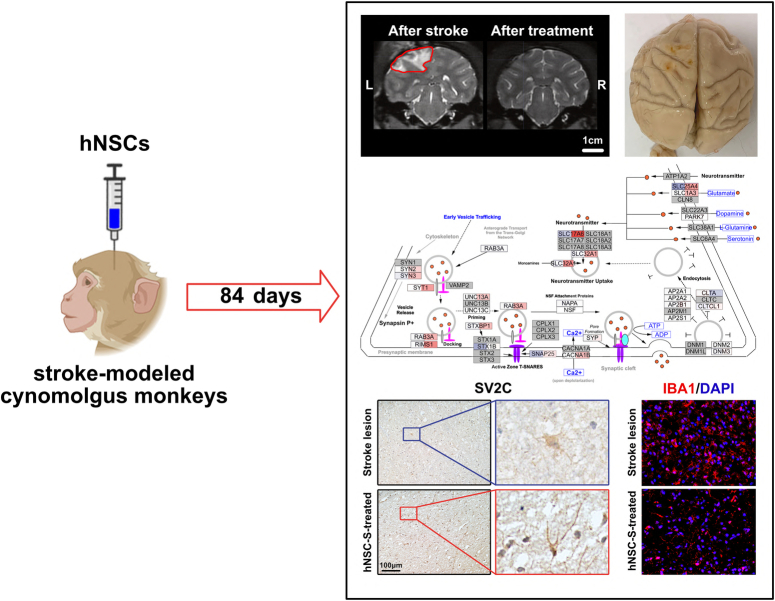

## Introduction

Highlights

- Neural stem cells accelerate neurological recovery safely after ischemic stroke.
- A possible mechanism is the regulation of post-stroke inflammatory responses.
- Activation of GABAergic neural transmission is found.
- Another possible mechanism is restoring the mitochondrial dysfunction.
- De-suppression of related extracellular vesicle micro-RNAs may be crucial.

Ischemic stroke is the most common neurovascular disorder and remains the leading cause of disability and death worldwide^1^. Intravenous fibrinolysis and mechanical thrombectomy remain the standard therapy for acute ischemic stroke^2^. However, both therapies are highly time-dependent. There is currently no effective medical treatment recommended for stroke patients when the time windows have been missed. Intracerebral transplantation of human neural stem cells (hNSCs) may be an effective surgical intervention for the treatment of ischemic stroke according to animal models^3,4^ and clinical trials^5,6^. In 2006, Roitberg *et al*.^7^ reported that hNSCs can survive 105 days and partly undergo neuronal differentiation in cynomolgus monkeys with ischemic stroke. Recently, PISCES study^5^ and PISCES-2 study^6^ demonstrated that intracerebral administration of hNSCs induced no adverse events and improved neurological functions in patients with chronic ischemic stroke.

The precise mechanisms underlying the effects of hNSC transplantation on ischemic stroke have not been fully elucidated. Proteomes can depict the status and dynamic changes of protein expression in different specimens, which is useful for the identification of the key pathways involved in a therapy^8^. Moreover, hypoxia can impact the release, the composition, and the function of micro-RNAs (miRNAs) in plasma-derived extracellular vesicles (EVs), which may further influence the neurogenesis and angiogenesis after stroke^9^. However, no previous studies demonstrate the alteration of the proteome and the plasma-derived EV miRNAs after NSC transplantation for ischemic stroke in non-human primates.

To evaluate the efficacy and safety of hNSCs for ischemic stroke, we transplanted hNSCs to cynomolgus monkeys 4 days after ischemic stroke and followed up for 84 days. Further, proteomic analysis and EV miRNA sequencing were performed to reveal the potential mechanism of hNSC therapy.

## Materials and methods

Full details of the materials and methods are shown in the Supplementary Document, Supplemental Digital Content 1, http://links.lww.com/JS9/C763. This work was reported in line with the ARRIVE criteria^10^, Supplemental Digital Content 2, http://links.lww.com/JS9/C764.

### Ethics statements

**Figure 1:**
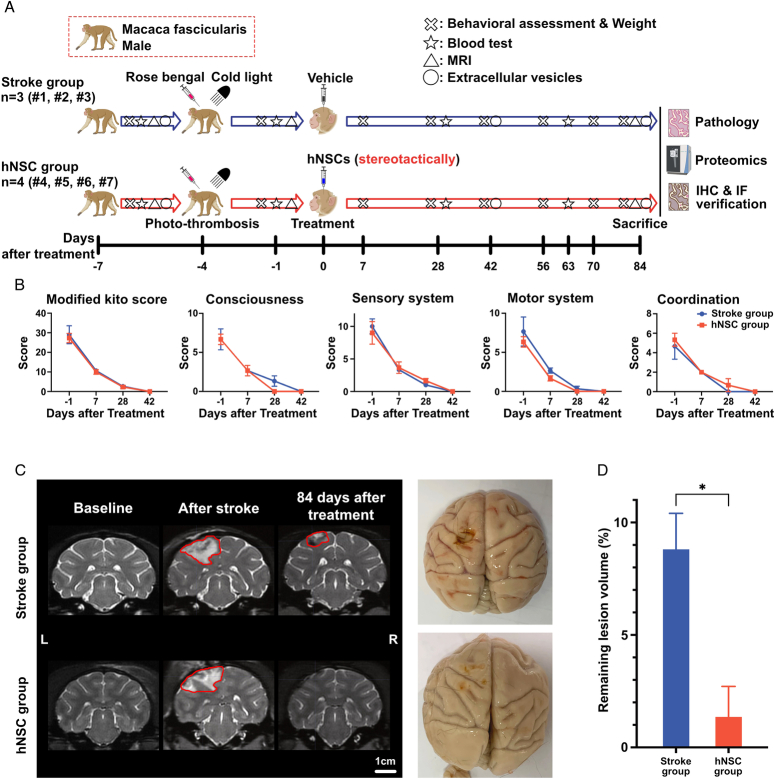
hNSCs accelerate neurological recovery in stroke-modeled *Macaca fascicularis.* (A) The workflow of this study. (B) Behavioral assessment with the modified Kito score in the stroke (*n*=3) and hNSC groups (*n*=4). (C, D) Remaining infarct lesion volume in the hNSC and stroke groups evaluated by T2-weighted MRI and the gross specimen of the brain. The remaining infarct lesion volume in the hNSC group was significantly smaller than the stroke group. *P<0.05. hNSC, human neural stem cell; IF, immunofluorescence; IHC, immunohistochemical staining; MRI, magnetic resonance imaging.

The experimental protocols were approved by the animal experiment committee and the ethics committee of Peking Union Medical College (approval number: XC19002). All experimental procedures were performed according to the Animal Welfare Act. Seven male cynomolgus monkeys (*Macaca fascicularis*) were housed in an environmentally controlled facility. The workflow of this study is shown in Figure 1A.

### Rose Bengal photothrombosis

The ischemic stroke model was induced by Rose Bengal photothrombosis^11,12^. In brief, the left precentral gyrus was infarcted by platelet aggregation induced by Rose Bengal (330000; Sigma-Aldrich, Darmstadt, Germany) and cold light (KL 1500 LCD; Schott, Mainz, Germany). General anesthesia was induced by an intramuscular injection of tiletamine and zolazepam (4 mg/kg; Zoletil). We then opened a skull window (15 mm×20 mm) at 15 mm lateral to the midline and the midpoint of the anteroposterior diameter. Five min after intravenous injection of Rose Bengal (50 mg/kg), the exposed cerebral cortex was irradiated with the light intensity of level 5 for 10 min, and this was repeated after a 5-min pause.

### hNSC transplantation

#### Preparation of hNSCs

Undifferentiated hNSCs were obtained from an NSC line derived from the human fetal forebrain^13^, which was established by the team of Professor Luan Zuo at the Sixth Medical Center of PLA General Hospital, China, and the quality of hNSCs was verified by the National Medical Products Administration of China (SH202001141). On the day of transplantation, hNSCs were dissociated and rewashed with normal saline (NS) before a final concentration adjustment.

### Cell transplantation

Seven cynomolgus monkeys were randomly assigned to two groups: (a) stroke group (*n*=3) and (b) hNSC group (*n*=4).

Four days after the induction of stroke, monkeys received the transplantation of hNSCs (hNSC group) or vehicle (stroke group). Monkeys in both groups were placed under deep anesthesia and then secured on a stereotactic frame (Stoelting, IL, USA) in the conventional stereotaxic position with the external auditory meatus and the inferior orbital margins in the same horizontal plane. After the incision, the skull windows that were used during modeling were exposed. A needle was inserted at the site based on coordinates (anterior–posterior, 0 mm, medial–lateral, 15 mm, relative to the midpoint of the anteroposterior diameter; dorsal–ventral, −10 mm, relative to the dura) to reach the left putamen^14^. For each monkey, ~2×10^6^ hNSCs in 100 µl NS or an equal volume of NS were transplanted using a micro syringe.

### Animal sacrifice

Monkeys in both groups were sacrificed 84 days (3 mo) after transplantation. Under deep anesthesia, animals were perfused intravenously through the superior vena cava with cold normal saline. Brains, livers, lungs, spleen, kidneys, and testicles were carefully removed. Brains were carefully dissected at 4°C. Different brain regions were then put on dry ice immediately and were stored at −80°C. The other organs were stored in 10% formaldehyde at 4°C.

### Behavioral assessment

Neurological function, which was assessed using the modified Kito scale^15,16^, and body weight were measured on days −7, −1, 7, 28, 42, 56, 63, 70, and 84 after transplantation. The Kito scale comprises 100 scores covering four components: consciousness (28 scores), sensory system (22 scores), motor system (32 scores), and coordination of skeletal muscles (18 scores). Higher scores indicate worse neurological function. The behavioral assessment was performed by two authors independently and disagreements were resolved by the senior authors.

### Magnetic resonance imaging

MRI was performed on days −7, −1, and 84 after transplantation using a 3.0-T MR scanner with an eight-channel human knee coil at the Centre for MRI Research, Peking University. High-resolution 3D-T1-weighed and 3D-T2-weighed images were obtained. The infarct volume was measured using ITK-SNAP software (v 3.8.0, http://www.itksnap.org/). The ratio between the lesion volume on day 84 and that after the stroke (the remaining infarcted lesion volume) was calculated and analyzed.

### Blood tests

In both groups, 10 ml venous blood samples were collected on days −7, −1, 28, and 63 after transplantation. Complete blood counts and levels of alanine aminotransferase (ALT), aspartate aminotransferase (AST), lactate dehydrogenase (LD), creatinine, alpha-fetoprotein (AFP), carcinoembryonic antigen (CEA), and CA129 were tested.

### Pathology

After careful dissection, the livers, lungs, kidneys, spleens, and testicles were fixed in formaldehyde (10%) at 4°C for 24 h. After dehydration by a series of graded alcohols, the tissues were embedded in paraffin and then cut into 5 µm-thick sections, which were stained with hematoxylin–eosin (H&E) staining and evaluated using an optical microscope.

### Immunohistochemical (IHC) staining and analysis

Using standard procedures, sections of frozen left precentral gyrus tissues were incubated with anti-vesicular glutamate transporter 2 (SLC17A6, 135403) or anti-synaptic vesicle glycoprotein 2C (SV2C, 119202; both from Synaptic Systems, Göttingen, Germany) antibodies, followed by staining with 3,3′-diaminobenzidine and hematoxylin solution. The positive, brown-colored signal for protein expression was quantified using Image-Pro Plus 6.0 software. The dendrite length measurement was determined using the NeuronJ plug-in (v1.4.3) in ImageJ software (v1.53, https://imagej.nih.gov/).

### Immunofluorescence (IF) staining and analysis

Frozen left precentral gyrus and hippocampus sections were incubated with anti-glial fibrillary acidic protein (GFAP, 16825-1-AP), anti-ionized calcium binding adaptor molecule 1 (IBA1, 10904-1-AP), anti-Nestin (19483-1-AP, all from Proteintech, Hubei, China), anti-doublecortin (DCX, 4604S; Cell Signaling Technology, Massachusetts), Anti-CD68 (sc-20060; Santa Cruz Biotechnology, Texas), Anti-NeuN (ab104224; Abcam, Cambridge, UK) antibodies. The nucleus was counterstained with the DAPI (4′,6-diamidino-2-phenylindole) solution. The branch length and soma area were analyzed with ImageJ software (v1.53, https://imagej.nih.gov/).

### Quantitative real-time reverse transcription PCR (qRT-PCR)

Total RNA from the left precentral gyrus tissues was extracted using TRIzol (15596018; Invitrogen). Reverse transcription was performed using the PrimeScript RT Master Mix Kit (RR036A; Takara). RNA expression was determined by qRT-PCR using TB Green Premix Ex Taq II (RR820A; Takara) on a Bio-Rad CFX96 system. Glyceraldehyde-3-phosphate dehydrogenase (GAPDH) served as reference genes. Relative gene expression was quantified using the 2^−ΔΔCt^ method. The primer information is listed in the Supplementary Document, Supplemental Digital Content 1, http://links.lww.com/JS9/C763.

### Proteome analysis

A tandem mass tag (TMT)-10-labeled proteome and a label-free proteome of the left and right precentral gyrus and hippocampus were performed as described previously^17–20^.

In the TMT-10-labeled proteomes, samples from the right precentral gyrus of the stroke group (intact control) were labeled with TMT 126 and 127N, while the samples from the left precentral gyrus of the stroke group (stroke lesion) were labeled with TMT 127C and 128N. Monkeys #3 and #7 were not used for proteomics. Specimens from the left precentral gyrus of three monkeys in the hNSC group (hNSC-S-treated) were labeled with TMT 128C, 129N, and 129C. After TMT labeling, high-performance liquid chromatography (HPLC) fractionation and liquid chromatography–tandem mass spectrometry (LC–MS/MS) was performed.

The proteome raw data were entered into Proteome Discoverer 2.2 (Thermo Fisher Scientific) and searched against *M. fascicularis* proteins in the FASTA database downloaded from UniProt (released on 6 November 2020). The detailed parameters used for the protein identification were shown in the Supplementary Document, Supplemental Digital Content 1, http://links.lww.com/JS9/C763.

After protein identification, the credible proteins were defined as unique peptides ≥2 and FDR ≤0.01. The Ensembl database (https://www.ensembl.org/) was used to map cynomolgus monkeys (*M. fascicularis*) proteins to human (*Homo sapiens*) proteins. Orthogonal partial least squares discriminant analysis (OPLS-DA) was performed with the ‘Wu Kong’ platform (https://www.omicsolution.com/wkomics/main/). Significantly changed proteins were identified using the following thresholds: downregulation, 0.833 (2^−0.263^) and upregulation, 1.200 (2^0.263^). Gene ontology (GO) analysis was performed using DAVID (https://david.ncifcrf.gov/) and Panther (http://www.pantherdb.org/), and SRplot (https://www.bioinformatics.com.cn) was used for GO visualization^21^. The STRING database (http://www.string-db.org) was used to predict protein–protein interaction networks. Pathway analysis was performed using the WikiPathway database of the WEB-based Gene SeT AnaLysis Toolkit (http://www.webgestalt.org/). All interaction networks were further visualized by Cytoscape (v3.9.1, https://cytoscape.org/).

The mass spectrometry proteomics data have been deposited to the ProteomeXchange Consortium (http://proteomecentral.proteomexchange.org) via the iProX partner repository^22^ with the dataset identifier PXD032871.

### EV extraction and miRNA sequencing

In both groups, a total of 12 ml venous peripheral blood was collected on days −7, 42, and 84 after transplantation. The plasma samples were centrifuged at 3000 rpm for 15 min at 4°C. The EVs were isolated by size exclusion chromatography. A transmission electron microscope (H-7650; Hitachi, Tokyo, Japan) was used to observe the morphology of isolated EVs. ZetaView PMX 110 (Particle Metrix, Meerbusch, Germany) equipped with a 405 nm laser was used to quantify the size and abundance of isolated EVs. Western blot with anti-CD63 (1:200, sc-5275, Santa Cruz, USA), anti-HSP90 (1:1000, 4877; Cell Signaling, USA), and anti-calnexin (1:500, 10427-2; Proteintech, USA) antibodies were used to confirm the extracted EVs.

Total RNA was extracted and purified from EV-enriched fractions using miRNeasy Mini Kit (Qiagen, Germany) following the manufacturer’s instructions. The extracted RNA was reverse transcribed with PrimeScript RT reagent Kit (Perfect Real Time, Takara Bio, USA) to synthesize complementary DNA. For small RNA libraries, QIAseq miRNA Library Kit (Qiagen, Germany) was performed for sequencing libraries with 1–500 ng RNA as input material per sample. Index codes were added to attribute sequences for each sample. Afterward, the library quality assessment was carried out using the Qsep100 Bio-Fragment Analyzer and qPCR. The index-coded samples were subsequently clustered on the Illumina cBot Cluster Generation System using TruSeq PE Cluster Kit v3-cBot-HS (Illumina, USA). After the clustering procedure, the libraries were sequenced on the Illumina novaseq6000 for paired-end reads generation.

The raw reads of small RNA were processed by filtering small nuclear RNA (snRNA), transfer RNA (tRNA), small nucleolar RNA (snoRNA), ribosomal RNA (rRNA), and other ncRNA with the software Bowtie2^23^. The remaining reads were then mapped to miRNA sequences from miRbase and *Macaca fascicularis*_5.0 Genome (GCA_000364345.1) to detect known miRNAs. MiRNA read counts were generated from the mapping results and normalized to transcripts per million (TPM). A threshold of TPM >5 was applied to filter out lowly expressed miRNAs.

The differentially expressed miRNAs were identified by the R package DESeq2 (1.36.0)^24^, with an absolute fold change >1.5 and the false discovery rate (FDR) <0.05, corrected by Benjamini–Hochberg adjustment. To assess the homology of miRNAs, we explore the data presented by Veeranagouda *et al*.^25^ to assess their conservativeness between *M. fascicularis* and *Homo sapiens*. For miRNA target identification, the R package multiMiR (2.3.0)^26^ was applied to find the target genes of miRNAs. In order to retrieve reliable miRNA–gene relationships, we select miRNA–gene pairs that exist in both validated datasets based on experimental results and predicted datasets based on prediction algorithms. To interrogate the biological functionalities of the enriched miRNAs, we conduct gene enrichment analysis for the miRNA target genes by the R package clusterProfiler (4.8.2)^27^. An FDR <0.05 was considered statistically significant for the pathway enrichment analysis.

Other R packages utilized for creating charts were as follows: heatmap plots were drawn by pheatmap (1.0.12). The volcano plots, bar plots, and bubble plots were created with ggplot2 (3.4.3)^28^. The miRNA–gene network plots were visualized with Cytoscape (v3.9.1, https://cytoscape.org/) with an organic layout.

### Statistical analysis

For the behavior tests and the clinical laboratory results, *t-*tests with Benjamini–Krieger–Yekutieli multiple comparison adjustments were used to determine the statistical significance of differences between groups. For IF analysis, one-way ANOVA followed by Tukey’s multiple comparison tests were used. *P*<0.05 was set as the threshold for statistical significance. Figures and the statistical analysis were constructed using GraphPad Prism software (v8.0; GraphPad, San Diego, CA).

## Results

### hNSCs accelerate neurological recovery in stroke-modeled *M. fascicularis*

After the ischemic stroke induced by Rose Bengal photothrombosis, the consciousness, sensory system, motor system, and coordination of the skeletal muscles were all affected in cynomolgus monkeys in the stroke and hNSC groups (Fig. 1B). In both groups, the modified Kito scores decreased over time, indicating the spontaneous recovery of neurological functions. However, there was a trend toward faster recovery in the hNSC group, especially in the consciousness dimension.

Eighty-four days after transplantation, T2-weighted MRI showed that the remaining infarcted lesion volume of the hNSC group was significantly smaller than that of the stroke group (1.3±2.3% vs. 8.8±2.8%, *P*=0.02) (Fig. 1C, D and Supplementary Fig. S1, Supplemental Digital Content 3, http://links.lww.com/JS9/C765).

### No remarkable side effects were produced from hNSC treatment

**Figure 2:**
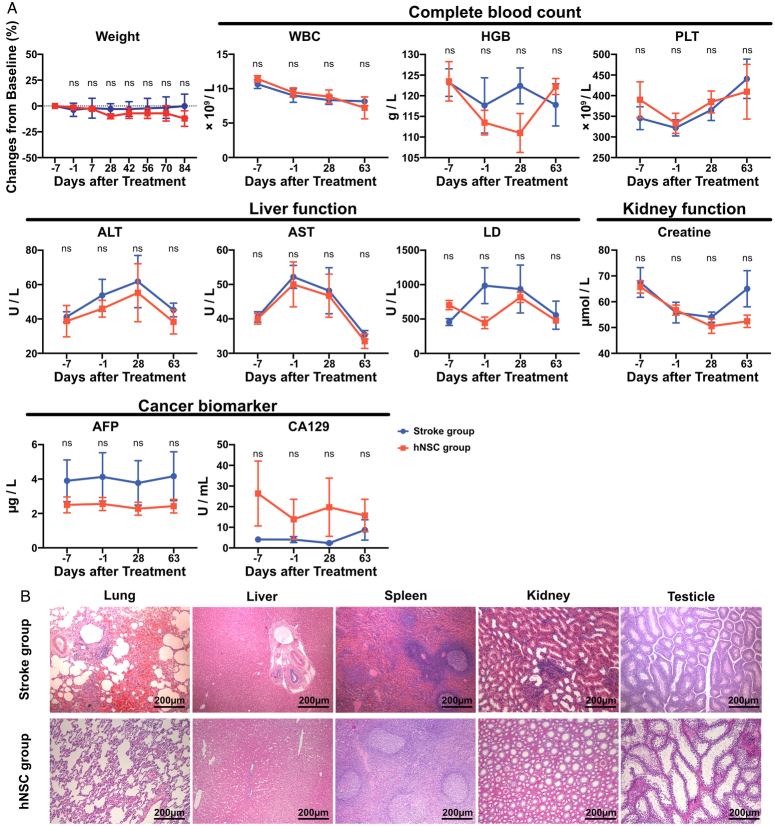
No remarkable side effects (myelosuppression, hepatic or renal impairment, or tumorigenesis) are found after hNSC treatment. (A) The weight changes, complete blood count, liver and kidney function, and cancer biomarkers of the hNSC and stroke groups. (B) Pathology of lungs, liver, spleen, kidney, and testicles indicate no neoformation 84 days after hNSC transplantation. AFP, alpha-fetoprotein; ALT, alanine aminotransferase; AST, aspartate aminotransferase; Cr, creatine; HGB, hemoglobin; hNSC, human neural stem cell; LD, lactate dehydrogenase; ns, no significant difference; PLT, platelet; WBC, white blood cell.

No significant differences were observed between the stroke and hNSC groups in weight changes, complete blood counts, liver function, or kidney function, indicating no myelosuppression or hepatic or renal impairment after hNSC treatment (Fig. 2A). In addition, there was no significant elevation of the cancer biomarker (AFP and CA129) in the hNSC group. H&E staining also confirmed that there was no neoformation of the major organs 84 days after hNSC transplantation (Fig. 2B and Supplementary Fig. S2, Supplemental Digital Content 4, http://links.lww.com/JS9/C766), which indicated that tumorigenesis did not occur after hNSC treatment.

### Protein profiles of the precentral gyrus of cynomolgus monkeys

**Figure 3:**
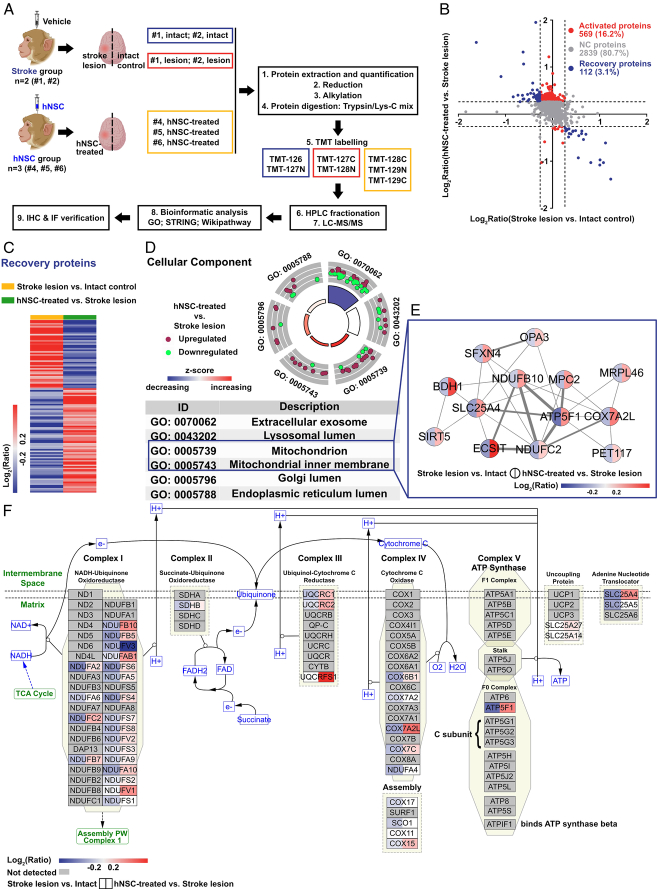
hNSC transplantation accelerated the neurological recovery by restoring the mitochondrial electron transport chain dysfunction in cynomolgus monkeys. (A) The workflow of the analysis of the proteomes generated from the left and right precentral gyrus of the hNSC and stroke groups. (B) The scatter diagram depicts the recovery proteins and activated proteins involved in hNSC therapy. Proteins that were significantly upregulated (≥2^0.263^) after stroke (stroke lesion vs. intact control; horizontal axis) and downregulated (≤2^−0.263^) after hNSC treatment (hNSC-S-treated vs. stroke lesion; vertical axis) and proteins which were downregulated after stroke and upregulated after hNSC treatment are defined as recovery proteins (blue dots). Proteins that were not significantly changed after stroke but significantly changed after hNSC treatment are defined as activated proteins (red dots). (C) The heatmap of the recovery proteins indicates that these proteins are significantly changed after stroke and restored after hNSC transplantation. (D) The cellular component domain of gene ontology analysis of the recovery proteins. Purple dots: upregulated proteins; green dots: downregulated proteins; red background: terms are mainly enriched by upregulated proteins; blue background: terms are mainly enriched by downregulated proteins. (E) Protein–protein interaction networks of the recovery proteins are enriched in the cellular component terms of the mitochondrion and the mitochondrial inner membrane. (F) Enrichment of the recovery proteins in the electron transport chain-oxidative phosphorylation system in the mitochondria pathway (WP111). The colors in the left and right half of each node indicate the ratio of stroke lesion vs. intact control and hNSC-treated vs. stroke lesion, respectively. Red background: overexpressed proteins; blue background: downregulated proteins.

The workflow of the proteome analysis generated from the left and right precentral gyrus of the hNSC and stroke groups is shown in Figure 3A. LC–MS/MS analysis revealed a total of 5051 credible proteins. Using the Ensembl database, these credible proteins were mapped to 3520 human proteins, which were used for further analysis. The protein abundance of the right (intact side) and left (lesion side) precentral gyrus in the stroke group was referred as the intact control and stroke lesion, respectively, whereas the abundance of the left precentral gyrus in the hNSC group was denoted as the hNSC-treated (Fig. 3A). According to the OPLS-DA (Supplementary Fig. S3, Supplemental Digital Content 5, http://links.lww.com/JS9/C767), the protein expression patterns of each sample from the same group were similar, while there were significant between-group differences.

**Figure 4:**
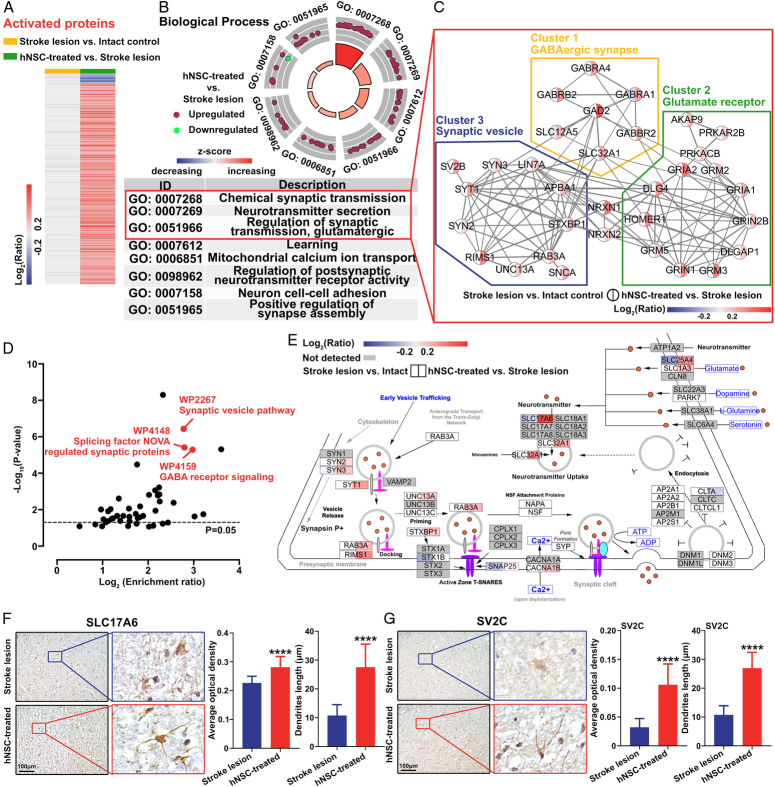
hNSC transplantation promotes GABAergic and glutamatergic neurogenesis and regulates the post-infarct inflammatory response in the infarct left precentral gyrus. (A) The heatmap shows that the activated proteins of the infarcted left precentral gyrus did not change significantly after stroke, and most were upregulated after hNSC transplantation. (B) Gene ontology analysis of the activated proteins shows that the activated proteins were mainly enriched in the synapse-related process of the biological process domain. Purple dots: upregulated proteins; green dots: downregulated proteins; red background: terms are mainly enriched by upregulated proteins; blue background: terms are mainly enriched by downregulated proteins. (C) Protein–protein interaction networks show three highly connected clusters. Cluster 1 (yellow circle) proteins are involved in GABAergic synaptic transmission. Cluster 2 (green circle) proteins are related to glutamate receptors. Cluster 3 (blue circle) proteins are related to the synaptic vesicle. (D) According to the WikiPathway database, activated proteins were mainly enriched in pathways related to synaptic transmission (red). (E) The synaptic vesicle pathway (WP2267) did not change significantly after stroke but was significantly activated after hNSC transplantation. The colors in the left and right half of each node indicate the ratio of stroke lesion vs. intact control and hNSC-treated vs. stroke lesion, respectively. Red background: overexpressed proteins; blue background: downregulated proteins. (F, G) Immunohistochemistry staining of the left precentral gyrus in the stroke and hNSC groups indicates that the synapse-specific proteins, vesicular glutamate transporter 2 (VGLUT2, also known as SLC17A6) (F) and synaptic vesicle glycoprotein 2C (SV2C) (G), were significantly upregulated in the hNSC group. A significant increase in the number and length of dendritic branches was also observed. ****P<0.0001.

The comparison of the stroke lesion with the intact control suggested alterations in the protein expression 3 months after stroke, while the comparison of the hNSC-treated left precentral gyrus with the stroke lesion indicated the altered pattern of protein expression after hNSC transplantation. As shown in Figure 3B, 67 (1.9%) proteins were downregulated after stroke and upregulated after hNSC treatment, while 45 (1.3%) proteins were upregulated after the stroke and downregulated after hNSC treatment. These 112 (3.1%) proteins were considered recovery proteins involved in the effects of hNSC treatment (blue dots). Besides, the expression of 569 (16.2%) proteins was not significantly changed after stroke but was differentially expressed after hNSC treatment. These proteins were considered activated proteins involved in the effects of hNSC therapy (red dots). The function of the recovery proteins (Fig. 3) and activated proteins (Fig. 4) were further analyzed, respectively.

### hNSC transplantation restored the mitochondrial energy metabolism

Recovery proteins were defined as proteins that were significantly changed after stroke and restored after hNSC transplantation (blue dots in Fig. 3B, C). GO analyses of recovery proteins indicated that, in the cellular component domain, the recovery proteins were significantly enriched in terms of mitochondrion and inner mitochondrial membrane (Fig. 3D). Besides, proteins enriched in these terms were mainly upregulated after hNSC transplantation (Fig. 3D). The protein–protein interaction networks of these proteins were further analyzed (Fig. 3E). In this highly connected network, proteins that were related to the mitochondrial energy metabolism, such as ATP synthase subunit b (ATP5F1) and NADH dehydrogenase [ubiquinone] 1 subunit C2 (NDUFC2), served as a hub, and these proteins were all downregulated during the stroke and upregulated after hNSC treatment. The WikiPathway database was used to illuminate the biological pathways related to the recovery proteins. Recovery proteins were significantly enriched in the electron transport chain-oxidative phosphorylation system in the mitochondria pathway (WP111, Fig. 3F). This pathway was predominantly downregulated after stroke and upregulated after hNSC treatment, suggesting that hNSC transplantation reversed the mitochondrial electron transport chain dysfunction of the infarcted precentral gyrus by rescuing the oxidative phosphorylation system.

Moreover, as shown in Figure 3D, many recovery proteins were also significantly enriched in the extracellular exosome, suggesting that the EVs might be crucial to the therapeutic effects of hNSCs. Thus, we performed the EV extraction and analysis as shown in Figure 7 (see below).

### hNSC transplantation promoted GABAergic and glutamatergic synaptogenesis

Activated proteins were defined as proteins that were not significantly changed after stroke but were significantly altered after hNSC transplantation (red dots in Fig. 3B), most of which (96.0%) were upregulated (Fig. 4A). Chemical synaptic transmission (6.5%), neurotransmitter secretion (2.3%), and regulation of synaptic transmission (glutamatergic) (1.8%) were the most enriched terms in the biological process domain (Fig. 4B). All proteins enriched in these terms were upregulated after hNSC transplantation (Fig. 4B). Consistently, in the cellular component domain, activated proteins were mainly involved in the glutamatergic synapse (10.6%), dendritic spine (5.3%), and axon (7.4%).

The protein–protein interactions of the activated proteins related to synapses were further analyzed. Figure 4C showed that none of the proteins in the network were significantly changed after stroke, but all were upregulated after hNSC treatment. Three highly connected clusters were formed in the network. Proteins in cluster 1 (yellow) were mainly involved in GABAergic synaptic transmission. Proteins in cluster 2 (green) were predominantly components of the glutamate receptors. Cluster 3 (blue) mainly contained synaptic vesicle-associated proteins. MS/MS spectrum data of SLC32A1 and GAD2 confirmed that both proteins were not significantly changed after stroke and significantly upregulated after hNSC transplantation (Supplementary Fig. S4, Supplemental Digital Content 6, http://links.lww.com/JS9/C768). According to the WikiPathway database (Fig. 4D), the activated proteins were predominantly enriched in pathways related to synaptic transmissions, such as the synaptic vesicle pathway (WP2267), which was not significantly changed after stroke, but significantly upregulated after hNSC treatment (Fig. 4E).

Immunohistochemical staining revealed two synaptic vesicle-specific proteins, SLC17A6^29^ and SV2C^30^, in the left precentral gyrus. SLC17A6 and SV2C are highly enriched in synaptic vesicles and are widely used as presynaptic markers^29,30^. In accordance with the proteome analysis, SLC17A6 (Fig. 4F) and SV2C (Fig. 4G) were significantly upregulated in the left precentral gyrus of the hNSC group compared with that of the stroke group. In addition, a significant increase in dendritic branch density and length was observed after hNSC treatment (Fig. 4F, G). These results suggested that hNSC treatment for ischemic stroke promotes synaptogenesis, especially of the GABAergic and glutamatergic synapses.

### hNSC transplantation alleviated neuroinflammation after stroke by suppressing resident glia activation and mediating peripheral immune cell infiltration

According to the proteome results, the expression levels of the astrocyte and microglia-specific protein, GFAP and IBA1, were significantly upregulated after stroke and downregulated after hNSC transplantation, suggesting an anti-neuroinflammatory effect of hNSCs. The response of the brain to ischemic injury comprises a sophisticated cascade of both acute and chronic inflammatory processes. This involves the secretion of pro-inflammatory cytokines, the rapid activation of resident glial cells including microglia and astrocytes, and the subsequent recruitment of a variety of peripheral inflammatory cells to the ischemic brain tissue^31^.

**Figure 5:**
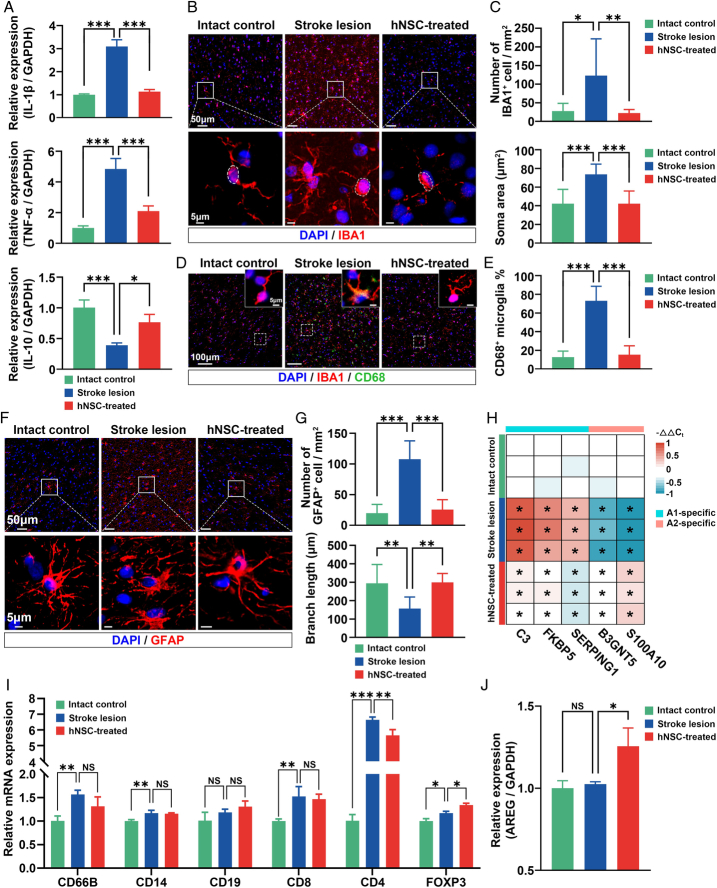
hNSC transplantation alleviates neuroinflammation in ischemic stroke lesions. (A) The mRNA expression of inflammatory factors (IL-1β and TNF-α) and anti-inflammatory cytokine (IL-10) in intact control, stroke lesion, and hNSC-treated group (*n*=3 per group). (B) Representative images of microglia (IBA1-positive) in the precentral gyrus of intact control, stroke lesion, and hNSC-treated group. (C) Graphs showing analysis and quantification of microglia number and their soma area (*n*=8 per group). (D) Representative double immunofluorescence staining for IBA1 (red) and CD68 (green) in intact control, stroke lesion, and hNSC-treated group. (E) Graph showing analysis of the percentage of CD68-positive microglia (*n*=8 per group). (F) Representative images of astrocyte (GFAP-positive) in the precentral gyrus of intact control, stroke lesion, and hNSC-treated group. (G) Graphs showing analysis and quantification of astrocyte number and their branch length (*n*=8 per group). (H) Heatmap of A1-specific and A2-specific astrocyte reactive transcripts in the precentral gyrus of intact control, stroke lesion, and hNSC-treated group. (I) The mRNA expression of CD66B, CD14, CD19, CD8, CD4, and FOXP3 in the precentral gyrus of intact control, stroke lesion, and hNSC-treated group (*n*=3 per group). (J) The mRNA expression of AREG in the precentral gyrus of intact control, stroke lesion, and hNSC-treated group (*n*=3 per group).

To further investigate whether hNSC transplantation alleviates the neuroinflammation caused by ischemic stroke, we first determined the levels of inflammatory cytokines in the stroke lesion with or without hNSC transplantation. We observed a notable increase in the levels of pro-inflammatory cytokines IL-1β and TNF-α alongside a significant decrease of the anti-inflammatory cytokine IL-10 in the stroke lesion by qRT-PCR (Fig. 5A). However, the expressions of these cytokines recovered after the hNSC transplantation (Fig. 5A), indicating the anti-inflammatory capabilities of hNSCs. Next, the effects of hNSCs on resident glial cells were investigated by IF. After a stroke, there was an observed increase in both the number and soma size of microglia, which characterized microglia activation^32^ (Fig. 5B, C). The activation was further confirmed by the elevation of CD68-positive microglia after stroke (Fig. 5D, E). In the hNSC-treated left precentral gyrus, there was a significant reduction of the activated microglia (Fig. 5B–E). Additionally, compared to the stroke lesion, the hNSC-treated area exhibited a significant alleviation in astrogliosis and a notable restoration in the shortening of astrocytic branches, suggesting that hNSC transplantation might alter the phenotype of astrocytes after stroke (Fig. 5F, G). According to a recent study, two different types of reactive astrocytes (A1 and A2) could be induced by neuroinflammation and ischemia. A1 exhibits neurotoxic effects through the release of pro-inflammatory mediators, whereas A2 confers neuroprotective effects by upregulating neurotrophic factors^33,34^. By measuring the expression of A1 (C3, FKBP5, and SERPING1) and A2 (B3GNT5 and S100A10)-specific markers, it was revealed that astrocytes exhibited an A1-like reactive signature in the stroke lesion, and they shifted towards an A2-like reactive astrocyte phenotype after hNSC treatment (Fig. 5H). These results suggested that hNSCs could suppress the activation of harmful resident glial cells to alleviate neuroinflammation.

Further analysis of peripheral immune cell markers CD66B (neutrophil), CD14 (monocyte/macrophage), CD19 (B cell), CD8 (cytotoxic T cell), CD4 (helper T cell), and FOXP3 (regulatory T cell) showed elevated levels in the stroke lesion (Fig. 5I). However, hNSC transplantation not only significantly reduced CD4+ T cell infiltration but also enhanced the infiltration of regulatory T cells in the stroke area (Fig. 5I), suggesting that hNSCs can mediate the peripheral immune cell infiltration. Regulatory T cells exert protective roles against post-stroke inflammation by producing the anti-inflammatory cytokine IL-10^35^, which was consistent with our observations of IL-10 upregulation after hNSC transplantation. Besides, regulatory T cells can release AREG to suppress astrogliosis and enhance brain recovery^36^. An upregulation of AREG was also detected in the hNSC-treated area (Fig. 5J), implying the mechanism by which hNSCs suppress astrogliosis.

### hNSC transplantation promoted neurogenesis of the hippocampus after stroke

**Figure 6:**
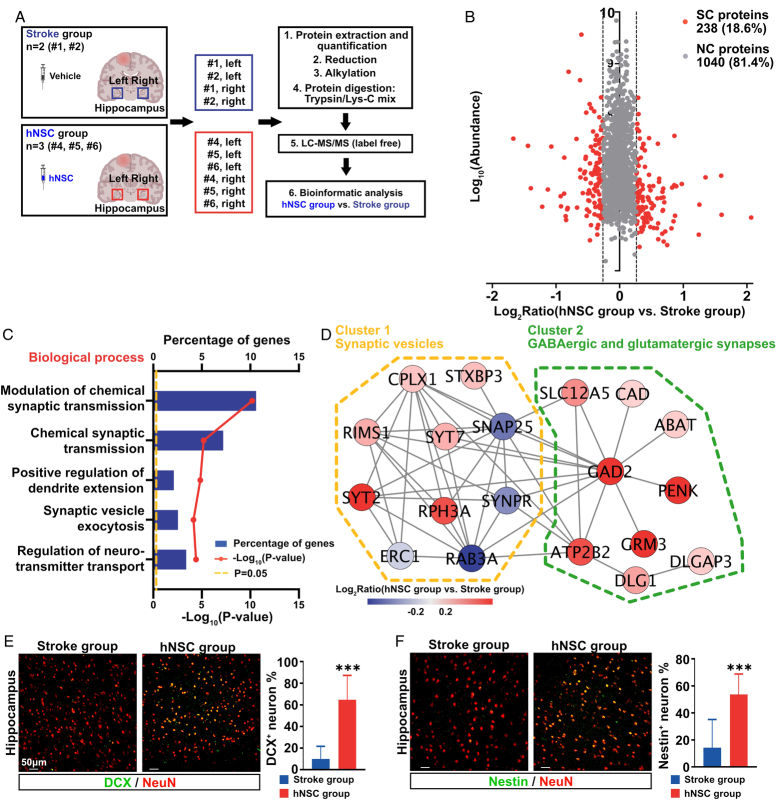
hNSC transplantation promotes neurogenesis in the hippocampus. (A) The workflow of the proteome analysis of the left and right hippocampus. (B) The scatter diagram depicts significantly changed proteins (red) in the hippocampus of the hNSC group compared to the stroke group post-transplantation. (C) Gene ontology analysis of the significantly changed proteins indicates that these proteins are predominantly enriched in the synaptic transmission category of the biological process domain. (D) Protein–protein interaction networks show two highly connected clusters. Cluster 1 (yellow) proteins are related to synaptic vesicles, most of which are upregulated after the hNSC treatment. Cluster 2 (green) proteins are mainly related to GABAergic and glutamatergic synapses, and all are upregulated after hNSC transplantation. (E) Representative double immunofluorescence staining for DCX (green) and NeuN (red) in the hippocampus of stroke group and hNSC group. Graph showing analysis of the percentage of DCX-positive neurons (*n*=8 per group). (F) Representative double immunofluorescence staining for Nestin (green) and NeuN (red) in the hippocampus of stroke group and hNSC group. Graph showing analysis of the percentage of Nestin-positive neurons (*n*=8 per group).

Analysis of the protein profiles of the hippocampus revealed 2081 credible proteins, and 1278 proteins were successfully mapped to the human database (Fig. 6A). For each protein, the average abundance on both sides of the hippocampus was calculated. A total of 238 (18.6%) proteins were significantly changed in the hNSC group compared with the stroke group (Fig. 6B). The significantly changed proteins were enriched in the biological process of synaptic transmission (Fig. 6C), and the cellular components of the glutamatergic synapse (10.5%) and synaptic vesicle (3.8%). The protein–protein interaction network of the proteins aggregated into two highly connected clusters (Fig. 6D). Proteins in cluster 1 (yellow) were related to synaptic vesicles, and cluster 2 (green) proteins were mainly related to GABAergic and glutamatergic synapses. Similar to the protein profile of the left precentral gyrus, these GABAergic and glutamatergic synapse-related proteins were all upregulated in the hippocampus after hNSC transplantation, indicating that hNSC transplantation could indirectly promote neurogenesis in the hippocampus. To confirm that neurogenesis was enhanced in the hippocampus, IF of neurogenesis markers, doublecortin (DCX) and Nestin, was performed, illuminating that, in hippocampus, the ratio of DCX-positive and nestin-positive neurons were both significantly elevated after hNSC transplantation (Fig. 6E, F).

### hNSC transplantation altered the levels of peripheral EV miRNA after strokes

As shown in Figure 3D, 38.7% of the recovery proteins were enriched in the extracellular exosome, indicating the critical role of EVs in hNSC therapeutic effects. Thus, for both groups, we extracted the EVs from the peripheral blood before stroke, 42 days and 84 days after transplantation. Transmission electron microscopy confirmed the presence of EV (Supplementary Fig. S5A, Supplemental Digital Content 7, http://links.lww.com/JS9/C769). Nanoparticle tracking analysis revealed that the isolated vesicles spanned a range of sizes from 50 to 250 nm (Supplementary Fig. S5B, Supplemental Digital Content 7, http://links.lww.com/JS9/C769). Besides, we confirmed that the isolated vesicles expressed CD63 and HSP90, which were markers of EV by western blotting, while they were calnexin-negative (Supplementary Fig. S5C, Supplemental Digital Content 7, http://links.lww.com/JS9/C769).

**Figure 7:**
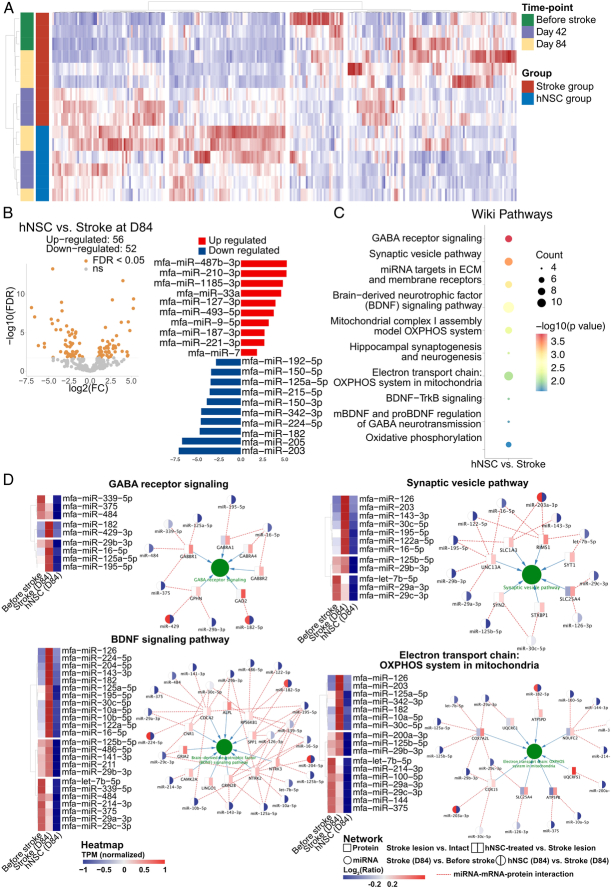
hNSC transplantation altered the level of EV micro-RNA in cynomolgus monkeys with ischemic stroke. (A) unsupervised hierarchal clustering analysis of the most variable 250 miRNAs. (B) Volcano plots depicting differentially expressed miRNAs and bar plots presenting the top 10 significantly upregulated and downregulated miRNAs of the hNSC group vs. the stroke group. (C) Dot plots showing top enriched Wiki pathways. (D) Heatmaps depicting miRNA expression patterns involved in the selected pathways before stroke and 84 days after transplantation in both groups with a miRNA–protein pathway network on the right of each heatmap. In the network, each circle represents a miRNA, and the colors in the left and right half indicate the ratio of stroke (D84) vs. before stroke and hNSC (D84) vs. stroke (D84), respectively; each rectangle represents a protein, and the colors in the left and right half of each node indicate the ratio of stroke lesion vs. intact control and hNSC-treated vs. stroke lesion, respectively.

Notably, miRNA serves as a crucial constituent of EV. miRNA extracting and sequencing were further performed. The extracted miRNA ranged from 19 to 25 bp, and most of them were 22 bp (Supplementary Fig. S5D, Supplemental Digital Content 7, http://links.lww.com/JS9/C769). Heatmap and unsupervised hierarchal clustering analysis showed that the EV miRNA was differentially expressed after stroke and after NSC treatment compared to the healthy control (Fig. 7A). To explore the influence of hNSCs on miRNA expression after stroke, we compare the expression of each miRNA between stroke group and hNSC group 12 weeks after treatment. A total of 108 miRNAs were significantly differentially expressed (Fig. 7B), including upregulated miR-9-5p.

Enrichment analysis of miRNA-targeted genes revealed the biological process of modulation of chemical synaptic transmission, regulation of neurotransmitter levels, and synapse organization (Supplementary Fig. S6, Supplemental Digital Content 8, http://links.lww.com/JS9/C770). In the cellular component domain, these genes were enriched in glutamatergic synapse and postsynaptic density. These results were consistent with our results of proteomic analysis. WikiPathway enrichment (Fig. 7C) showed the alerted miRNA may regulate GABA receptor signaling (*P*=1.67E−04), synaptic vesicle pathway (*P*=4.46E−04), BDNF signaling pathway (*P*=2.03E−03), and electron transport chain: OXPHOS system in mitochondria pathway (*P*=8.14E−03). Notably, all of the altered miRNAs enriched in these pathways were downregulated in the hNSC groups. Besides, according to the proteomic data of the precentral gyrus, the miRNA-targeted-gene-translated proteins were mainly upregulated after hNSC transplantation (Fig. 7D). Since miRNAs mainly function as inhibitors of their targeted mRNAs, the results of miRNA sequencing and proteome were consistent, which suggested that the activation of the pathways could be a result of de-suppression of their related miRNAs after hNSC transplantation.

## Discussion

In this study, we evaluated the therapeutic efficacy and safety of stereotactically intracerebral transplantation of hNSCs for ischemic stroke and revealed the potential mechanism in cynomolgus monkeys for the first time. Our results provide evidence that hNSC transplantation accelerates neurological recovery after ischemic stroke without remarkable side effects. In our analysis of proteomes of the precentral gyrus and hippocampus and the EV miRNA sequencing, we found that hNSC transplantation promoted GABAergic and glutamatergic neurogenesis and attenuated post-infarct inflammatory responses in both the ischemic infarcted left precentral gyrus and hippocampus. hNSCs transplantation also reversed the mitochondrial electron transport chain dysfunction in the infarct lesion. Further, miRNA-sequencing suggested that the direct and indirect therapeutic effects of hNSCs might be achieved through the regulation of EVs and miRNA.

### hNSCs can promote neurogenesis

One hypothesis of brain repair with NSC is that transplanted stem cells enhance endogenous repair mechanisms activated after cerebral ischemia. Andres *et al*.^37^. transplanted hNSCs into rat cortex 7 days after ischemic stroke and observed that hNSCs significantly promoted dendritic remodeling in both sides of the cortex, together with stem cell-induced functional recovery. They also demonstrated that axonal transport was inhibited after stroke and was rescued by hNSCs, thus facilitating neurological recovery in ischemic stroke survivors. In our study, we identified activated proteins in the infarcted left precentral gyrus that were significantly enriched in the biological process of synaptic transmission, especially the GABAergic and glutamatergic synapses and the synaptic vesicle pathway. Immunohistochemical staining confirmed significant upregulation of synaptic vesicle-specific proteins, SLC17A6 and SV2C^29,30^, after hNSC treatment, as well as a significant increase in dendritic branch density and length. These results demonstrated that hNSC treatment promotes neurogenesis in the infarct region after ischemic stroke.

### hNSCs may attenuate post-stroke inflammatory responses and activate GABAergic neural transmission

Inflammatory responses are key processes in the pathogenesis of ischemic stroke^38,39^. hNSC transplantation could significantly reduce the post-stroke inflammatory response and suppress the activation of microglia^40^. Other previous studies also suggested that stem cells attenuated harmful immune and inflammatory responses by releasing immune regulatory factors, such as nitric oxide (NO), transforming growth factor-beta (TGF-β), and interleukin (IL)-10^39,41^, which was consistent with our results. Besides, a shift from an A1-like astrocyte toward an A2-like astrocyte was revealed after hNSC transplantation. According to a recent study, A1 astrocytes could significantly increase the expression of several classical complement cascade genes, which were destructive to synapses and harmful to neurogenesis. Conversely, A2 astrocytes showed neural protective potential by upregulating many neurotrophic factors^33,34^. Thus, the anti-inflammatory effects of hNSC might be a key factor in the treatment of stroke.

GABA also has modulatory effects on the glial and immune cells in the post-ischemic brain. Kuhn *et al*.^42^ found that microglia expressed GABA receptors, and the pro-inflammatory reaction of microglia was attenuated by the activation of GABA receptors. Lee *et al*.^43^ demonstrated that, after stimulation with the commonly used inflammatory stimulants lipopolysaccharide and interferon-γ, GABA suppressed the immune response of astrocytes and microglia, resulting in a reduced release of nuclear factor kappa B (NF-κB), and the inflammatory cytokines, tumor necrosis factor (TNF)-α and IL-6, which might contribute to ischemic neuronal damage^44^. Thus, GABA exerts protective effects on neurons by regulating the inflammatory response after ischemic stroke. Our results indicated the expression of GAD2, GABA type A receptors, GABA type B receptors, SLC32A1, and SLC12A5 was activated after hNSC transplantation in the infarct left precentral gyrus. GAD2 is a key isoform of glutamic acid decarboxylase, which catalyzes the synthesis of GABA^45^ and is mainly responsible for synaptic GABA production during neurotransmission^46^. SLC32A1 is a vesicular GABA-specific transporter that is involved in the loading of GABA into the synaptic vesicles^47^. SLC12A5 is required for neuronal chloride homeostasis as a potassium-chloride cotransporter, which is fundamental to GABAergic neural transmission^48^. Thus, the activation of these proteins indicated that hNSCs promoted GABAergic neurogenesis after ischemic stroke.

According to previous studies and our results, hNSCs may exert regulatory effects on post-stroke immune and inflammatory responses via the activation of GABAergic neural transmission, which further accelerates brain repairs after ischemic stroke.

### hNSCs may indirectly improve the survival of stroke-induced endogenetic neurons by regulating the inflammatory responses in the hippocampus

Our results indicated that neurogenesis was significantly enhanced in the hippocampus after hNSC transplantation. Besides, the significantly changed proteins of the hippocampus were predominantly related to GABAergic and glutamatergic synapses and synaptic vesicles, indicating that the hNSC transplantation may have indirect effects on other brain areas.

Besides the infarct region, neuronal death also occurs in the distal brain areas, such as the hippocampus, after the ischemic stroke^49^. According to recent studies, the dentate gyrus of the hippocampus is a region where new neurons are continuously generated during a lifetime^50^, which is fundamental for the maintenance of hippocampal memory^51^. In pathological conditions, the proliferative response of precursor cell subpopulations in the dentate gyrus is significantly increased during the first week after ischemic stroke^49,52^. However, approximately 80% of stroke-induced endogenetic neurons die during the first 2–6 weeks post-ischemia^52^. Conceivably, the uncontrolled inflammatory response accompanying the ischemic damage may be the chief culprit for the poor survival of the newly generated hippocampal neurons^53,54^. Administration of the anti-inflammatory drugs has been shown to reduce the number of activated microglia^55^ and increase the number of neuroblasts^56^ and new mature neurons^55^ in the hippocampus. According to our results, hNSCs alleviated neuroinflammation after stroke, which might also benefit the hippocampus. Thus, we extrapolate that hNSC transplantation improves the survival of stroke-induced endogenetic neurons by regulating the inflammatory response after ischemic stroke, thereby promoting neurogenesis in the hippocampus.

### hNSCs reverse the mitochondrial electron transport chain dysfunction after ischemic stroke

Recent studies focused on the roles of mitochondria in ischemia-related neuronal death, which include reactive oxygen species generation and scavenging, electron transport chain dysfunction, apoptosis, mitochondrial dynamics, and inflammation^57^. Preservation and recovery of mitochondrial function are fundamental for cell survival and neurogenesis after ischemic stroke^57^. Our results suggested that proteins related to mitochondrial energy metabolism served as a hub cluster in the protein–protein interaction networks of recovery proteins, suggesting that mitochondrial energy metabolism was impaired after ischemic stroke and restored after hNSC transplantation.

### hNSCs might also exert their direct and indirect therapeutic effects by regulating EV miRNAs

In the central nervous system, numerous miRNAs are intricately linked to neuroprotection following an ischemic stroke by fine-tuning the expression of their target genes^58^. According to a previous study, miR-9 assumes a crucial role in promoting neuronal survival and regeneration after stroke. The transfection of miR-9 mimic can enhance the viability of neuronal cells after ischemia, fostering increased proliferation and neurite elongation, which collectively contributes to the recovery process after stroke^59^. Our finding revealed that after the transplantation of hNSCs, miR-9-5p, a member of the miR-9 family, was significantly upregulated in the EV of the peripheral blood. Besides, we conducted a joint analysis of brain proteome and EV miRNA data. The target genes of the significantly changed miRNAs were enriched in numerous pathways, such as GABA receptor signaling, synaptic vesicle pathway, and electron transport chain, which were also found in the proteomic results. Notably, the protein expression related to these pathways was mainly upregulated after hNSC transplantation, while the miRNA levels were downregulated. These findings suggested that the activation of the pathway could be a result of de-suppression of their related miRNAs. Collectively, these results indicated that hNSCs might exert their therapeutic effects through the transmission of EV miRNAs. This may also elucidate how the hNSCs regulated the hippocampus, which was not the directly transplanted site.

### Study limitations

Some limitations of our studies should be noted. First, the symptoms of the monkeys induced by Rose Bengal photothrombosis were relatively mild. Thus, no distinct differences in the results of behavior tests between the stroke and hNSC groups were observed 3 months after treatment. Indeed, according to previous studies^11^, the behavioral manifestations of ischemic stroke induced by Rose Bengal photothrombosis in non-human primates recovered gradually, and the behavior score 28 days after the operation was similar to that recorded before the operation, indicating strong spontaneous recovery of monkeys. Most of the previous studies on an ischemic stroke used a middle cerebral artery occlusion model in mice and rats. However, this method has a high mortality rate due to the huge damage to the animals. Therefore, we chose to employ the Rose Bengal photothrombosis model in this study. Besides, only a limited number of cynomolgus monkeys were available for use in our study, and thus, the results of this study should be interpreted with caution.

## Conclusions

Our study demonstrates that stereotactically, intracerebral transplantation of hNSC accelerates neurological recovery without remarkable side effects (myelosuppression, hepatic or renal impairment, or tumorigenesis) in cynomolgus monkeys after ischemic stroke. NSCs may promote neurogenesis by attenuating post-stroke inflammatory responses by suppressing resident glia activation, mediating peripheral immune cell infiltration, and activating GABAergic neural transmission in both the infarct region and the hippocampus. hNSC may also reverse mitochondrial electron transport chain dysfunction after ischemic stroke. Furthermore, the EV miRNAs may play a crucial role in the treatment of ischemic stroke with hNSCs. Further clinical trials are needed to confirm the clinical benefits of hNSCs in patients.

## Ethical approval

The experimental protocols were approved by the animal experiment committee and the ethics committee of Peking Union Medical College (approval number: XC19002).

## Consent

Not applicable.

## Source of funding

This work was supported by the National Key Research and Development Program of China (2018YFA0108602), the National Natural Science Foundation of China (82170799), the CAMS Initiative for Innovative Medicine (2021-1-I2M-019), the National High Level Hospital Clinical Research Funding (2022-PUMCH-C-042), the China Postdoctoral Science Foundation (2023M732450, 2023TQ0223, GZB20230478), and STI2030-Major project (2021ZD0201100).

## Author contribution

Y.-F.L., H.-T.L., C.-X.Y., R.-Z.W., and X.-J.B.: conceptualization; Y.-F.L., H.-T.L., and C.C.: data curation; Y.-F.L., H.-T.L., C.C., X.W., and W.G.: formal analysis; Y.-F.L. and X.-J.B.: funding acquisition; Y.-F.L., H.-T.L., C.C., C.-X.Y., and X.-N.L.: investigation and visualization; Y.-F.L., H.-T.L., C.C., and C.-X.Y.: methodology; W.G., R.-Z.W., and X.-J.B.: project administration; W.G. and X.-J.B.: resources; W.G., R.-Z.W., and X.-J.B.: supervision; Y.-F.L. and H.-T.L.: writing – original draft; C.C., C.-X.Y., X.-N.L., X.W., W.G., R.-Z.W., and X.-J.B.: writing – review and editing.

## Conflicts of interest disclosure

The authors declare no conflicts of interest related to this study.

## Research registration unique identifying number (UIN)

Not applicable.

## Guarantor

Xin-Jie Bao.

## Data availability statement

The mass spectrometry proteomics data have been deposited to the ProteomeXchange Consortium (http://proteomecentral.proteomexchange.org) via the iProX partner repository with the dataset identifier PXD032871. All other data used to support the findings of this study are available from the corresponding author upon request.

## Provenance and peer review

Not commissioned, externally peer-reviewed.

## Supplementary Material

**Figure:**
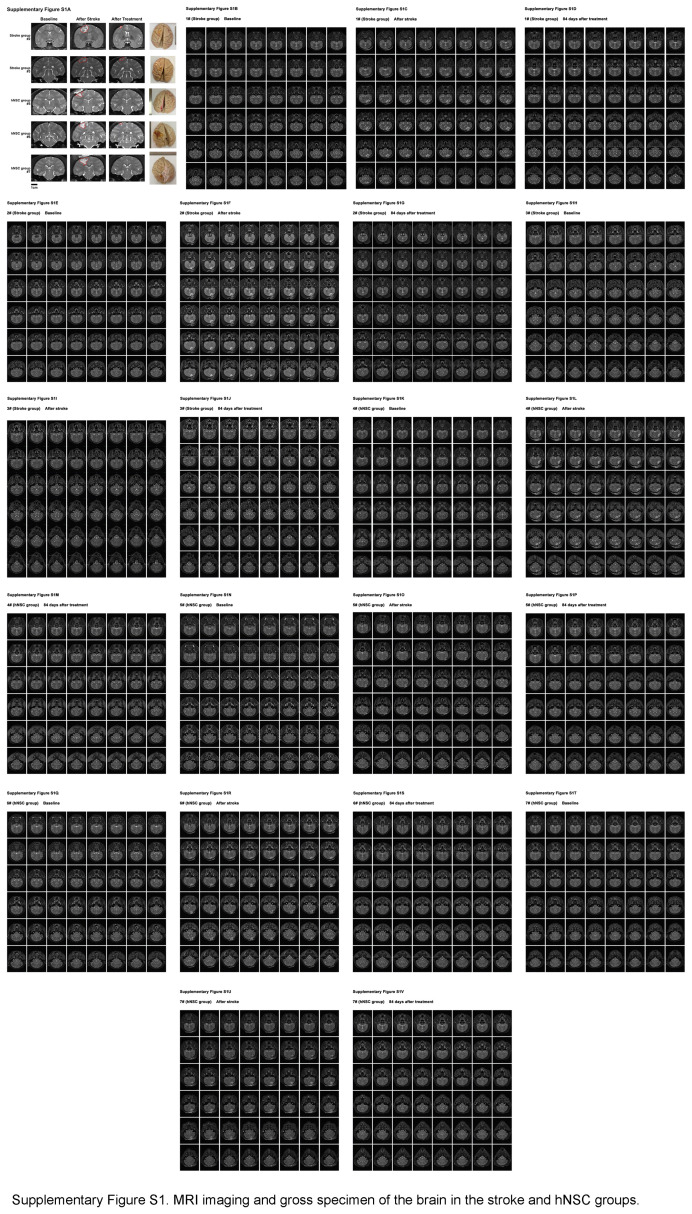


**Figure:**
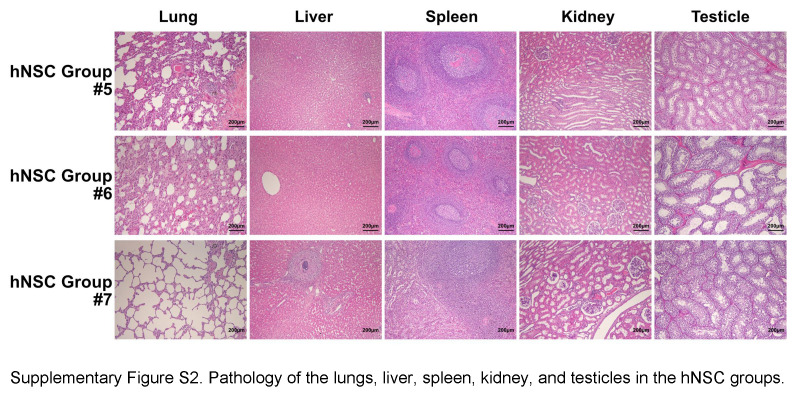


**Figure:**
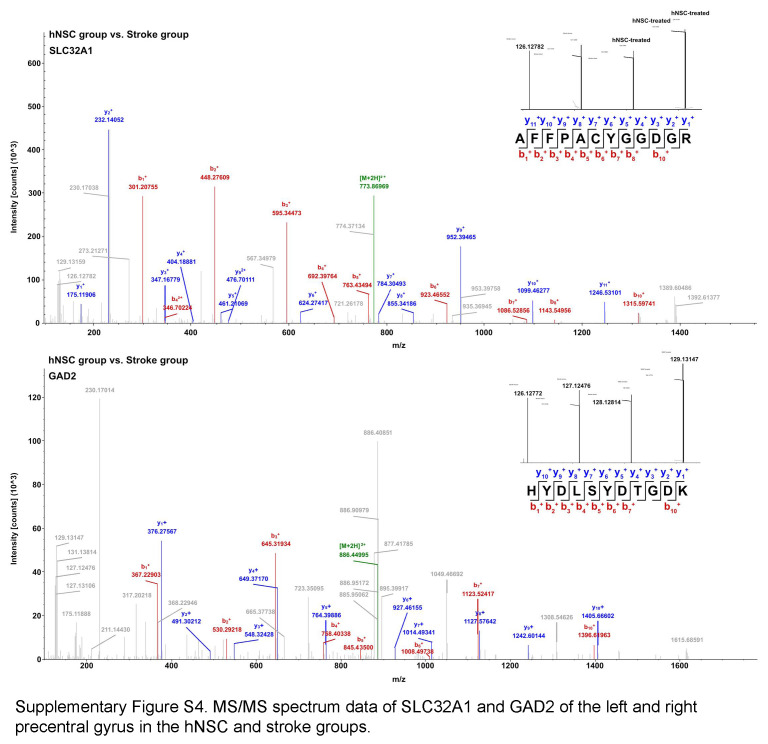


**Figure:**
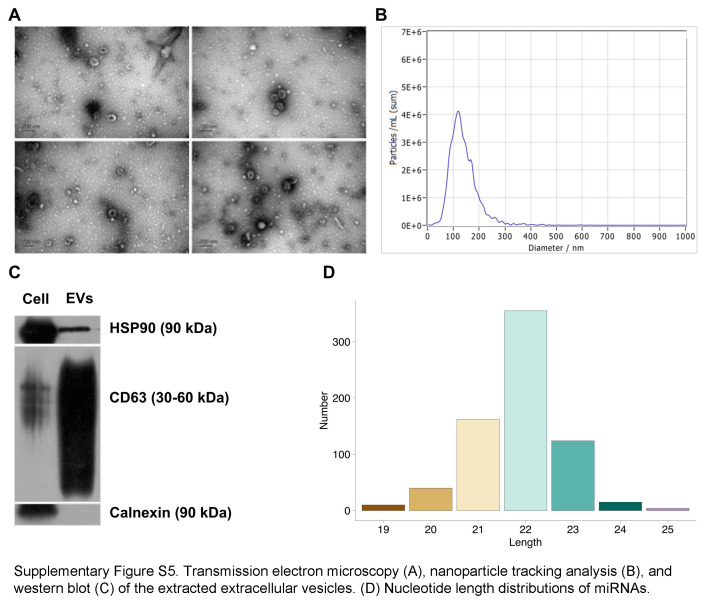


**Figure:**