# Supplementary material for: Stereotactically intracerebral transplantation of neural stem cells for ischemic stroke attenuated inflammatory responses and promoted neurogenesis: an experimental study with monkeys
Source: Int J Surg. 2024 Jun 14;110(9):5417–33. doi: 10.1097/JS9.0000000000001791 (PMC11392141; doi:10.1097/JS9.0000000000001791)
Supplement: Supplementary file 1 [file js9-110-5417-s001.pdf]

Supplementary Document. Detailed materials and methods

Ethics Statements

The experimental protocols were approved by the animal experiment committee and the ethics committee (approval number: XC19002). All experimental procedures were performed according to the Animal Welfare Act. Seven male cynomolgus monkeys (*Macaca fascicularis*) were housed in an environmentally controlled facility (22 ± 1 °C temperature; 50 ± 5% relative humidity; and 12 h light/12 h dark cycle with lights on at 7:00) and were fed at the same time (7:30, 12:00, 15:00). The workflow of this study is shown in Figure 1A.

Reagents and antibodies

Rose Bengal (330000) was purchased from Sigma-Aldrich (Darmstadt, Germany). Zoletil® (tiletamine and zolazepam) was purchased from Virbac (Carros, France). Iodoacetamide (IAA, I2273), dithiothreitol (DTT, D9163), triethylammonium bicarbonate buffer (TEAB, T7408), hydroxylamine solution (467804), Triton X-100 (V900502) and urea were purchased from Sigma–Aldrich (Burlington, VT, USA). Proteinase inhibitor cocktail and sequencing-grade trypsin/Lys-C (V5071) were purchased from Promega (Madison, WI, USA). The TMT 10-plex™ Isobaric Label Reagent Sets (90110), TMT 6-plex™ Isobaric Label Reagent Sets (90061) and Trifluoroacetic Acid (TFA, 85183, LC-MS Grade) were purchased from Thermo Fisher Scientific (Waltham, MA, USA). Antirabbit HRP-DAB Two Step IHC Detection Kit (PV-9001) and 3,3′-diaminobenzidine (ZLI-9019) were purchased from ZSGB-BIO (Beijing, China). Anti-CD68 antibody (sc-20060) was obtained from Santa Cruz Biotechnology (Texas). Anti-NeuN antibody (ab104224) was obtained from Abcam. Anti-doublecortin antibody (4604S) was obtained from Cell Signaling Technology (Massachusetts). Anti-SLC17A6 (also named VGLUT2, 135403) and anti-SV2C (119202) antibodies were obtained from Synaptic Systems (Göttingen, Germany). Anti-GFAP (16825-1-AP), anti-IBA1 (10904-1-AP), and anti-

Nestin (19483-1-AP) antibodies were obtained from Proteintech (Hubei, China). Cy3 conjugated Goat Anti-Rabbit IgG (GB21303), EDTA antigen retrieval solution (pH 8.0, G1206), BSA (G5001), DAPI (G1012), spontaneous fluorescence quenching reagent (G1221), and anti-fade mounting medium (G1401) were purchased from Servicebio (Hubei, China). Optimal Cutting Temperature Compound was purchased from Sakura Finetek (Tokyo, Japan). Adhesion microscope slides were purchased from Citotest (Jiangsu, China). Hematoxylin solution (G1120) and neutral balsam (G8590) were obtained from Solarbio (Beijing, China).

Rose Bengal photo-thrombosis

The ischemic stroke model was induced by Rose Bengal photo-thrombosis(1, 2). In brief, the left precentral gyrus was infarcted by platelet aggregation induced by Rose Bengal and cold light (KL 1500 LCD, Schott, Mainz, Germany). Firstly, general anesthesia was induced by an intramuscular injection of tiletamine and zolazepam (Zoletil®, 4mg/kg). Secondly, we opened a skull window of 15 mm × 20 mm at 15 mm lateral to the midline and a midpoint of the anteroposterior diameter. Five min after intravenous injection of Rose Bengal (50mg/kg), the exposed cerebral cortex was irradiated with the light intensity of level 5 for 10 min. Then, after 5 min pause, we irradiated them for another 10 min. The radiation field was kept within 38 degrees by dripping saline and a fan blower.

hNSC Transplantation

Preparation of hNSCs

Undifferentiated human neural stem cells (hNSCs) were obtained from an NSC line derived from the human fetal forebrain which was established by the team of Professor Luan Zuo at the Sixth Medical Center of PLA General Hospital, China(3), and the quality of hNSCs was verified by the National Medical Products Administration of China (SH202001141). Briefly,

after being fully informed according to the guidelines approved by the Ethics Committee of the Sixth Medical Center of PLA General Hospital, the forebrain tissue of legally terminated human embryos was isolated and the primary dissociated single-cell suspensions were incubated in serum-free NSC medium (Angecon Biotech). Primary cells were cultured at a density of 1×10^6cells/mL to form neurospheres at 37ºC with 5% CO_2. The neurospheres were digested with accutase (A1110501, Gibco, NY, USA) and replated every 7–10 days. On the day of transplantation, hNSCs were dissociated and rewashed before a final concentration adjustment.

Cell Transplantations

Seven monkeys were randomly assigned to two groups: (a) 4 monkeys as the hNSC group, (b) 3 monkeys as the stroke group. Monkeys received the transplantation of hNSCs or vehicle 4 days after stroke.

Under deep anesthesia, monkeys were secured on a stereotactic frame (Stoelting, IL, USA) in the conventional stereotaxic position with the external auditory meatus and the inferior orbital margins in the same horizontal plane. After the incision, the skull windows used during modeling were exposed. A needle was inserted at the site based on coordinates (AP, 0 mm, ML, 15 mm, relative to the midpoint of the anteroposterior diameter; DV, -10 mm, relative to the dura) to reach the left putamen(4). For each monkey, approximately 2×10^6 hNSCs in 100μL NS or an equal volume of NS was transplanted by a micro syringe at the left putamen. After 2 min, the needle was slowly withdrawn.

Animal Sacrifice

All monkeys were sacrificed 84 days (3 months) after transplantation. Under deep anesthesia, animals were perfused intravenously through superior vena cava with cold normal saline.

Brains, livers, lungs, spleen, kidneys, and testicles were carefully removed. Brains were carefully anatomized at 4ºC. Different brain regions were then put on dry ice immediately and were stored in a -80ºC freezer later. The other organs were stored in 10% formaldehyde at 4ºC.

Behavioral Assessment

Modified Kito scale(5) and the bodyweight measurement were performed on days -7, -1, 7, 28, 42, 56, 63, 70, and 84 after transplantation to evaluate the neurological function of monkeys. Kito scale (100 scores, with higher scores indicating worse neurological function) contains four components: consciousness (28 scores), sensory system (22 scores), motor system (32 scores), and coordination of skeletal muscles (18 scores). The behavioral assessment was performed by two authors separately. The disagreement about scores was confirmed by the senior authors.

Magnetic Resonance Imaging

Magnetic resonance imaging (MRI) was scanned on day -7, -1, and 84 after transplantation with 3.0-T MR scanner (Discovery MR750; GE Healthcare) with 8-channel human knee coil at the Centre for MRI Research, Peking University. High-resolution 3D-T1-weighed and 3D-T2-weighed images were obtained (140 sagittal slices, TR = 5.9 ms, TE = 2.9 ms, FA = 12°, FOV = 115 mm × 115 mm, matrix size = 192 × 192, ST = 0.6 mm, voxel size = 0.6 × 0.6 × 0.6 mm, number of excitations = 4, acquire time = 10 minutes and 32 seconds). The infarct volume was measured using ITK-SNAP software (v 3.8.0, http://www.itksnap.org/). The ratio between the lesion volume on day 84 and that before transplantation (the remaining infarcted lesion volume) was calculated and analyzed.

Clinical Laboratory Tests

A total of 10 ml venous blood samples were collected on days -7, -1, 28, and 63 after transplantation. Complete blood count, alanine aminotransferase (ALT), aspartate aminotransferase (AST), lactate dehydrogenase (LD), creatinine, alpha-fetoprotein (AFP), carcinoembryonic antigen (CEA), and CA129 were tested using Chemistry Analyzers AU6800 Series of Beckman Coulter® (Brea, CA, USA).

Pathology

After careful anatomy, the livers, lungs, kidneys, spleens, and testicles were fixed in formaldehyde (10%) at 4ºC for 24 hours. After dehydration by a series of graded alcohols, the tissues were embedded in paraffin and then cut into 5μm-thick slices which were used for hematoxylin-eosin (H&E) staining. The specimens were checked by an optical microscope.

Immunohistochemical Staining and Analysis

Frozen left precentral gyrus tissues were embedded in Optimal Cutting Temperature Compound (OCT), by placement on carbon dioxide dry ice. Tissues were retrieved from mold and then stored at -80°C overnight. Tissue-containing OCT blocks were sectioned in a cryostat (Thermo Cryotome E) and cut into 6 μm-thick slices, which were adhered onto adhesion microscope slides. The slides were rinsed by 0.01M PBS to wipe off OCT. Tissues on slides were permeabilized by 0.3% Triton X-100 for 30 minutes and then blocked using Anti-Rabbit HRP-DAB Two Step IHC Detection Kit reagent 1 for 30 minutes at room temperature before incubating with the primary antibody anti-SLC17A6 and anti-SV2C (1:200) in PBS overnight at 4°C. After washing in PBS, slides were incubated independently with reagents 2 and 3. After that, 3,3′-diaminobenzidine and Hematoxylin solution were used for visualization and nuclear localization respectively. Finally, the slides were then gradient dehydrated and sealed with neutral balsam. The tissue slides were captured with Olympus BX61 at brightfield under 20x

lens. The positive, brown-colored signal was extracted and quantified using Image-Pro Plus 6.0 software. The dendrite length measurement was finished by the NeuronJ plug-in (v1.4.3) in ImageJ software (v1.53, https://imagej.nih.gov/).

Immunofluorescence and Analysis

Generally, the frozen slides of the left precentral gyrus and hippocampus were immersed in EDTA antigen retrieval buffer (pH 8.0) and incubated with 3% BSA to block non-specific binding for 30 min. Throw away the blocking solution slightly and incubate slides with anti-GFAP and anti-AIF1 antibody (1:100, diluted with PBS) overnight at 4ºC. After being washed with PBS, slides were incubated with Cy3 conjugated Goat Anti-Rabbit IgG at room temperature for 50 min in dark conditions. After washing three times with PBS 5 min each, we incubate the slides with DAPI solution for 10 min. After being washed with PBS, slides were incubated with spontaneous fluorescence quenching reagent for 5 min and washed with water. Throw away liquid slightly, then coverslip with anti-fade mounting medium. Fluorescent microscopy (Nikon Eclipse C1, Nikon, Tokyo, Japan) was used to detect and collect images by UV excitation (for GFAP or IBA1, excitation 510-560 nm and emission 590 nm; for DAPI, excitation 330-380 nm and emission 420 nm).

Quantitative real–time reverse transcription PCR (qPCR)

Total RNA from cynomolgus monkeys left precentral gyrus tissues was extracted using TRIzol (Invitrogen, 15596018), according to manufacturer instructions. Reverse transcription was performed using the PrimeScript RT Master Mix Kit (Takara, RR036A). RNA expression was determined by qRT–PCR using TB Green Premix Ex Taq II (Takara, RR820A) on a Bio-Rad CFX96 system. GAPDH served as reference genes; relative gene expression was quantified using the 2-ΔΔCt method.

Primer information:

Primer name	Sequence (5' to 3')
IL1B-F	CGGTTGTTGTAGCCATGGAG
IL1B-R	GCAGTGCAGTGATCGTACAG
TNFA-F	GTCAACCTCCTCTCTGCCAT
TNFA-R	CCAAAGTAGACCTGCCCAGA
C3-F	CTCTCTGGCTGTCAACCTCA
C3-R	CGAGATGAGAACAAAGGCCG
FKBP5-F	CGAGTTTGAGTCAGCCAAGG
FKBP5-R	CCCATCGCTTTATTGGCCTC
SERPING1-F	CTTCTGCCCAGGACCTGTTA
SERPING1-R	GGCAATGCTGAATGGGGAAA
B3GNT5-F	GCCTCGCTACCAGTACTTGA
B3GNT5-R	CATTCAGCTGAGACCGAACG
S100A10-F	CATTTCACAAATTCGCGGGG
S100A10-R	CCATCTCTGCACTGGTCCA
IL10-F	GCCAAGCCTTGTCTGAGATG
IL10-R	AAGAAATCGATGACAGCGCC
AREG-F	CTCAGGCCATTATGCTGCTG
AREG-R	CCAGGTATTTGCGGTTCGTT
CD14-F	TAGACCTCAGCCACAACTCG
CD14-R	GTCTGTTGCAGCTGAGATCG
CD66b-F	ATATCTCAGGGCCTGCAGAC
CD66b-R	CCGGATGTACGCTGAATTGG
CD4-F	CTGACCCTGACCTTGGAGAG
CD4-R	GGAAAGCTAGCACCACGATG
CD8A-F	GCCCCACCTTCCTCCTATAC
CD8A-R	GCGGCTGAAGTACATGATGG
CD19-F	GAACGTGCTATCTCTCCCCA
CD19-R	CCCCTTCCTCTTCTTCTGGG
FOXP3-F	CTTTCACCTATGCCACGCTC
FOXP3-R	AACTCCAACTCATCCACGGT
GAPDH-F	CATCACTGCCACCCAGAAGACTG
GAPDH-R	CATCACTGCCACCCAGAAGACTG

Proteomes analysis

Protein preparation

Two sets of proteomes analyses were performed (Figure 3A, and Figure 5A). The left and right precentral gyrus and hippocampus from the hNSC group and the stroke group were carefully

dissected and stored under -80ºC cryopreservation before use. About 200mg of tissues were separated from each region. 8M urea in 0.1M phosphate-buffered saline (PBS) containing proteinase inhibitors was used for further tissue lysate preparation. After removing tissue debris by centrifugation at 12,000 rpm for 15 min at 4 °C, the protein concentration was determined by Nanodrop 2000 (Thermo Scientific).

TMT-labeled proteomes

Proteins from the left and right precentral gyrus of monkeys underwent TMT-10 labeling. 100μg proteins for each sample were prepared for TMT-labeling as described previously(6). Proteins were processed with 10 mM DTT for 30 min, followed by 25 mM IAA treatment (placed in the dark) for 30 min at room temperature. After dilution with PBS, proteins were digested by trypsin/Lys-C mix overnight at 37°C. To inactivate enzyme activity, the digested peptides were heated at 60°C for 30 min, and the solution was modulated to pH 1–2 with TFA. Next, Reverse-phase column chromatography (Oasis HLB, WAT094225, Waters) was used to desalt peptides, followed by vacuum concentration. The peptide powder was dissolved in 50 μl 200 mM TEAB, for further Tandem Mass Tag (TMT) labeling.

In the first set of proteomes, TMT 10-plex kits were used in this study. Samples from the right precentral gyrus of the stroke group (intact) were labeled with TMT 126 and 127N, while the samples from the left precentral gyrus of the stroke group (stroke lesion) were labeled with TMT 127C and 128N. Specimens from the left precentral gyrus of the 3 monkeys in the hNSC group (hNSC-treated) were labeled with TMT 128C, 129N, and 129C. After 1 h incubation at room temperature, 5% hydroxylamine was added for 15 min to terminate the reaction. The TMT-labeled peptides were then mixed, desalted, dried, and finally dissolved in 100 μl of 0.1% TFA for high-performance liquid chromatography (HPLC) fractionation.

The peptides were fractionated by gradient elution using an HPLC system (UltiMate™ 3000, Thermo Fisher Scientific) cooperated with an Xbridge BEH300 C18 column (1.0 ml/min, 4.6 × 250 mm, 2.5 mm, Waters) as described previously(6). Gradient elution buffer consists of phase A (ddH_2O, PH=10) and phase C (98% acetonitrile in ddH_2O, PH=10). The flow rate was set as 1.0 ml/min for 75min. Starting at 2 min, a total of 47 fractions were collected at a 1.5 min interval and were dried under vacuum. Peptides were then dissolved in 20 µl 0.1% TFA for LC-MS/MS analysis.

Peptides were fractionated for further LC-MS/MS analysis using the Orbitrap Fusion™ mass spectrometers (Thermo Fisher Scientific). The labeled peptides were loaded into UltiMate 3000 RSLCnano System (Thermo Fisher Scientific) equipped with a silica capillary column (75 µm ID, 150 mm length; Upchurch, Oak Harbor, WA, USA) packed with C-18 resin (300 Å, 2 µm; Varian, Lexington, MA, USA). Monkey precentral gyrus-derived peptides from each sample were achieved with a 120-min gradient elution using a 0.60 µl/min flow rate. The flowthrough peptides were ionized with the mass spectrometer in a positive-ion mode. Data were acquired using the directly interfaced Orbitrap Fusion mass spectrometer (operated in the data-dependent acquisition mode) with a single full-scan mass spectrum (350–1,350 m/z, 120,000 resolution) followed by data-dependent MS/MS scans (3 s) in an Ion Routing Multipole at 38% normalized collision energy (HCD).

Label-Free Proteomes

In the second set of proteomes, proteins extracted from the left and right hippocampus of the hNSC group and stroke group were processed for label-free proteomes. Protein preparation was the same as mentioned before. A total of 100µg proteins of each sample were treated with

DTT followed by the IAA process in dark. Trypsin/Lys-C mix digestion was in progress overnight at 37°C. Then, after inactivating the reaction, acidification, desalination, and vacuum centrifugation were performed in order.

LC-MS/MS proteomic analyses were performed with UltiMate 3000 RSLCnano System (Thermo Fisher Scientific), which interacted with the Fusion^{TM} mass spectrometer system (Thermo Fisher Scientific). Monkey hippocampus-derived peptides from each sample were achieved with a 120-min gradient elution using a 0.30 μl/min flow rate. A single full-scan mass spectrum (300–1,400 m/z, 120,000 resolution) was used, followed by data-dependent MS/MS scans (3 s) in an Ion Routing Multipole at 30% normalized collision energy (HCD).

Protein identification and quantification

TMT-labeled raw data and label-free raw data were entered into Proteome Discoverer 2.2 (Thermo Fisher Scientific) and searched against Macaca fascicularis protein FASTA database downloaded from UniProt (released on November 6, 2020). The SEQUEST-HT algorithm is executed for further search and the criteria are shown below: a maximum of two missed cleavages allowed; for TMT-10plex-labeled data, precursor mass tolerance and fragment mass tolerance are set at 10 ppm and 0.02 Da respectively, while 10 ppm and 0.6 Da for label-free data; static modifications include TMT10plex on the peptide N terminal (only for TMT-labeled data) and carbamidomethylation on C; dynamic modifications include oxidation on M and acetylation on N-Terminus of proteins; protein identification was considered valid when at least one peptide was detected with a false discovery rate (FDR) of less than 1%. All other parameters were set as default.

Bioinformatics analysis

After protein identification, the credible proteins were defined as unique peptides ≥ 2 and a false discovery rate ≤ 0.01. The Ensembl database (https://www.ensembl.org/) were used to map the proteins of cynomolgus monkeys (*Macaca fascicularis*) to the proteins of human (*homo sapiens*). Significantly changed (SC) proteins were identified using the following thresholds: downregulation, 0.833 ($2^{-0.263}$) and upregulation, 1.200 ($2^{0.263}$). Gene ontology (GO) analysis was performed using DAVID (https://david.ncifcrf.gov/) and Panther (http://www.pantherdb.org/), and SRplot (https://www.bioinformatics.com.cn) was used for GO visualization(7). The STRING database (http://www.string-db.org) was used for predicting protein-protein interaction networks. Pathway analysis was performed using the Wiki pathway database of the WEB-based Gene SeT AnaLysis Toolkit (http://www.webgestalt.org/). All interaction networks were further visualized by Cytoscape (v3.9.1, https://cytoscape.org/).

The mass spectrometry proteomics data have been deposited to the ProteomeXchange Consortium (http://proteomecentral.proteomexchange.org) via the iProX partner repository with the dataset identifier PXD032871.

Plasma collection and extracellular vesicle (EV) isolation

In both groups, a total of 12 ml venous peripheral blood was collected in in ethylenediaminetetraacetic acid (EDTA) tubes for 3 biological replicates (4 ml each) on days -7, 42, and 84 after transplantation. The plasma samples were centrifuged at 3000 rpm for 15 minutes at 4°C and then stored at -80°C before use. The EVs were isolated by size exclusion chromatography. Briefly, 1 mL of 0.8 μm-filtered plasma was 1.5-fold diluted in phosphate-buffered saline (PBS) and further purified using Exosupur® columns (Echobiotech, China). The samples were then eluted with 0.1 M PBS, and a total of 2 mL eluate fractions were collected according to the manufacturer's protocol. Subsequently, fractions were concentrated

to 200 µL through a 100 kDa cut-off Amicon® Ultra spin filters (Merck, Germany).

Transmission Electron Microscopy

A total of 10µL of the EV-enriched solution was placed on a copper grid, and negative staining with uranyl acetate was performed to enhance contrast. The morphology of isolated EVs was observed and photographed using a transmission electron microscope (H-7650, Hitachi, Tokyo, Japan).

Nanoparticle Tracking Analysis (NTA)

ZetaView PMX 110 (Particle Metrix, Meerbusch, Germany) equipped with a 405 nm laser was used to quantify the size and abundance of isolated EVs. The range of measurement is between 10 nm and 3 µm. A movie of 60 seconds at 30 frames per second was captured, and data acquisition and processing were carried out using NTA software (ZetaView 8.02.28).

Western Blot Analysis

The EV-enriched solution was denatured in 5× sodium dodecyl sulfonate (SDS) buffer and then subjected to western blot assay using standard procedures (10% SDS-polyacrylamide gel electrophoresis and 50 µg protein/lane). The primary antibodies used for EV characterization were anti-CD63 (1:200, sc-5275, Santa Cruz, USA), anti-HSP90 (1:1000, 4877, Cell Signaling, USA), and anti-calnexin (1:500, 10427-2, Proteintech, USA). The proteins were then imaged with the automatic chemiluminescence imaging analysis system Tanon4600 (Tanon, Shanghai, China).

RNA Extraction and Library Preparation

Total RNA was extracted and purified from EV-enriched fractions using miRNeasy® Mini Kit

(Qiagen, Germany) following the manufacturer's instructions. The extracted RNA was reverse transcribed with PrimeScript™ RT reagent Kit (Perfect Real Time, Takara Bio, USA) to synthesize complementary DNA. We used SMARTer Stranded Total RNA-Seq Kit (Takara Bio USA, Inc.) to construct long RNA sequencing libraries with a total amount of 250pg to 10ng RNA per sample as input material, and added index codes to annotate sequences for each sample. For small RNA libraries, QIAseq miRNA Library Kit (Qiagen, Germany) was performed for sequencing libraries with 1ng to 500ng RNA as input material per sample. Index codes were added to attribute sequences for each sample. Afterward, the library quality assessment was carried out by the Qsep100 Bio-Fragment Analyzer and qPCR. The index-coded samples were subsequently clustered on the Illumina cBot Cluster Generation System using TruSeq PE Cluster Kit v3-cBot-HS (Illumina, USA). After the clustering procedure, the libraries were sequenced on the Illumina novaseq6000 for paired-end reads generation.

Micro RNA (miRNA) analysis

The raw reads of small RNA were processed by filtering small nuclear RNA (snRNA), transfer RNA (tRNA), small nucleolar RNA (snoRNA), ribosomal RNA (rRNA), and other ncRNA with the software Bowtie2 (8). The remaining reads were then mapped to miRNA sequences from miRbase and *Macaca fascicularis*_5.0 Genome (GCA_000364345.1) to detect known miRNAs. MiRNA read counts were generated from the mapping results and normalized to transcripts per million (TPM). A threshold of TPM >5 was applied to filter out lowly expressed miRNAs.

The differentially expressed miRNAs were identified by the R package *DESeq2* (1.36.0) (9), with an absolute fold change > 1.5 and the false discovery rate (FDR) < 0.05, corrected by Benjamini-Hochberg adjustment. To assess the homology of miRNAs, we explore the data

presented by Veeranagouda *et al.* (10) to assess their conservativeness between *Macaca fascicularis* and *Homo sapiens*. For miRNA target identification, the R package *multiMiR* (2.3.0) (11) was applied to find the target genes of miRNAs. In order to retrieve reliable miRNA-gene relationships, we select miRNA-gene pairs that exist in both validated datasets based on experimental results and predicted datasets based on prediction algorithms. To interrogate the biological functionalities of the enriched miRNAs, we conduct gene enrichment analysis for the miRNA target genes by the R package *clusterProfiler* (4.8.2) (12). A FDR < 0.05 was considered statistically significant for the pathway enrichment analysis.

Other R packages utilized for creating charts were as follows: heatmap plots were drawn by *pheatmap* (1.0.12). The volcano plots, bar plots, and bubble plots were created with *ggplot2* (3.4.3). The miRNA-gene network plots were visualized with *visNetwork* (2.1.2).

Statistical analysis

The expression level of two target proteins between two groups in the immunohistochemical analysis was shown as average optical density quantified by Image-Pro Plus 6.0 software. The dendrite length was counted by the NeuronJ plug-in in ImageJ software. Data were presented as the mean ± standard deviation and *t-tests* were used to determine statistical significance. A *P*-value of less than 0.05 was considered statistical significance. The statistical figures were drawn with GraphPad Prism software (v8.0, GraphPad, San Diego, CA, USA).

1. Ikeda S, Harada K, Ohwatashi A, Kamikawa Y, Yoshida A, Kawahira K. A new non-human primate model of photochemically induced cerebral infarction. PLoS One. 2013;8(3):e60037.
2. Dietrich WD, Watson BD, Busto R, Ginsberg MD, Bethea JR. Photochemically induced cerebral infarction. I. Early microvascular alterations. Acta Neuropathol. 1987;72(4):315-25.
3. Luan Z, Liu W, Qu S, Du K, He S, Wang Z, et al. Effects of neural progenitor cell transplantation in children with severe cerebral palsy. Cell Transplant. 2012;21 Suppl 1:S91-8.
4. Szabo J, Cowan WM. A stereotaxic atlas of the brain of the cynomolgus monkey (Macaca fascicularis). J

Comp Neurol. 1984;222(2):265-300.
5. Kito G, Nishimura A, Susumu T, Nagata R, Kuge Y, Yokota C, et al. Experimental thromboembolic stroke in cynomolgus monkey. Journal of Neuroscience Methods. 2001;105(1):45-53.
6. Jia Y, Wang X, Chen Y, Qiu W, Ge W, Ma C. Proteomic and Transcriptomic Analyses Reveal Pathological Changes in the Entorhinal Cortex Region that Correlate Well with Dysregulation of Ion Transport in Patients with Alzheimer's Disease. Molecular neurobiology. 2021;58(8):4007-27.
7. Tang D, Chen M, Huang X, Zhang G, Zeng L, Zhang G, et al. SRplot: A free online platform for data visualization and graphing. PLoS One. 2023;18(11):e0294236.
8. Langmead B, Salzberg SL. Fast gapped-read alignment with Bowtie 2. Nat Methods. 2012;9(4):357-9.
9. Love MI, Huber W, Anders S. Moderated estimation of fold change and dispersion for RNA-seq data with DESeq2. Genome Biology. 2014;15(12):550.
10. Veeranagouda Y, Rival P, Prades C, Mariet C, Léonard JF, Gautier JC, et al. Identification of microRNAs in Macaca fascicularis (Cynomolgus Monkey) by Homology Search and Experimental Validation by Small RNA-Seq and RT-qPCR Using Kidney Cortex Tissues. PloS one. 2015;10(11):e0142708.
11. Ru Y, Kechris KJ, Tabakoff B, Hoffman P, Radcliffe RA, Bowler R, et al. The multiMiR R package and database: integration of microRNA-target interactions along with their disease and drug associations. Nucleic Acids Res. 2014;42(17):e133.
12. Wu T, Hu E, Xu S, Chen M, Guo P, Dai Z, et al. clusterProfiler 4.0: A universal enrichment tool for interpreting omics data. Innovation (Camb). 2021;2(3):100141.
